# A causal mediation model of ischemia reperfusion injury in the retina

**DOI:** 10.1371/journal.pone.0187426

**Published:** 2017-11-09

**Authors:** Maha Soliman, Kalina Andreeva, Olfa Nasraoui, Nigel G. F. Cooper

**Affiliations:** 1 Department of Anatomical Sciences and Neurobiology, University of Louisville, Louisville, KY, United States of America; 2 Department of Computer Engineering and Computer Science, University of Louisville, Louisville, KY, United States of America; University of Edinburgh, UNITED KINGDOM

## Abstract

The goal of this study is to develop a model that explains the relationship between microRNAs, transcription factors, and their co-target genes. This relationship was previously reported in gene regulatory loops associated with 24 hour (24h) and 7 day (7d) time periods following ischemia-reperfusion injury in a rat’s retina. Using a model system of retinal ischemia-reperfusion injury, we propose that microRNAs first influence transcription factors, which in turn act as mediators to influence transcription of genes via triadic regulatory loops. Analysis of the relative contributions of direct and indirect regulatory influences on genes revealed that a substantial fraction of the regulatory loops (69% for 24 hours and 77% for 7 days) could be explained by causal mediation. Over 40% of the mediated loops in both time points were regulated by transcription factors only, while about 20% of the loops were regulated entirely by microRNAs. The remaining fractions of the mediated regulatory loops were cooperatively mediated by both microRNAs and transcription factors. The results from these analyses were supported by the patterns of expression of the genes, transcription factors, and microRNAs involved in the mediated loops in both post-ischemic time points. Additionally, network motif detection for the mediated loops showed a handful of time specific motifs related to ischemia-reperfusion injury in a rat’s retina. In summary, the effects of microRNAs on genes are mediated, in large part, via transcription factors.

## Introduction

The proper function of the retina is associated with gene regulation, which is accomplished, in part, by activation or suppression of genes not only in a time specific manner but also in coordination with the expression of many other genes. Studying the coordinated gene expression requires an in-depth understanding of the interactions between genes and their regulators at the molecular level. In recent years, many models of gene regulatory networks combining transcription factors (TFs) and microRNAs (miRNAs) have been investigated [1–25]. The core of constructing these models revolves around three basic steps [1– 4]. The first step is to construct a miRNA-mediated gene or TF-mediated gene regulatory network that reflects the interactions between its entities via some experimental data. Depending on the mediated regulatory network, interactions can either occur between miRNAs-mRNAs, miRNAs-TFs, and TFs-mRNAs in a miRNA-mediated gene network or TFs-mRNAs, TFs-miRNAs, and miRNAs-mRNAs in a TF-mediated gene network. However, sometimes the limited knowledge of TFs-miRNAs in literature makes the miRNA-mediated gene network a prevailing approach. The second step is to translate the constructed network into a coherent analytical framework (mathematical or statistical) that can explain the interactions between the network entities. The third step is to characterize the model parameters by simulation or by supporting information from an existing database, or from the literature.

Statistical modeling has had a considerable share in modeling the regulatory network information for TFs and miRNAs and their target genes in many conditions and/or diseases, but largely in studies related to cancer [5–7]. A wide spectrum of approaches with different levels of complexity that dealt with various types of cancer was reported. For example, [8–11] used relatively simple statistical approaches based on correlation to address miRNA-mRNA networks associated with colorectal and pancreatic cancer respectively. In the context of prostate cancer [12], a classifier was used for exploiting almost every aspect of extractable information from mRNA/miRNA expression data of prostate tumor and normal samples. The classifier was used to detect numerous known and novel miRNA-mediated deregulated loops and networks in the disease. In glioma [13], a network-based method was used to construct an miRNA-mRNA regulatory network from combining paired expression profiles of 160 Chinese glioma patients. In glioblastoma [14], miRNA-mRNA was integrated with TF-mRNA regulatory information to generate one regulatory network for the disease. Statistical modeling was also used in drug identification such as experiments [15–17], in which the goal from constructing feed-forward loops of miRNA-TF-mRNA was to identify drug repurposing candidates in the context of Cystic Fibrosis (CF).

Mathematical modeling of the tertiary relations between miRNA-TF-mRNA has proven its usefulness in unraveling the role of miRNA-mediated network motifs in fine-tuning gene expression [18]. For example, mathematical modeling revealed that intercellular networks are particularly enriched with miRNA-TF-mRNA motifs that enable regulatory features such as homeostasis, oscillatory behavior, and all-or-nothing gene expression patterns [19]. In another model, these motifs were hypothesized to control gene expression programs at a temporal scale [20]. Additionally, these motifs were found to be vital for cell fate, including cell proliferation and apoptosis [21]. Recently, an increasing number of TF-miRNA circuits have been identified as having the structure of feed-backward loops (FBLs). These loops were found to give rise to bi-stability in gene expression, a sophisticated regulatory condition in which the network switches to a new state upon a transient perturbation, and to confer robustness to biological processes [22–24]. In this context, we recall the remarkable case of multiple TF-miRNA FBLs, which appear in the regulation of the E2F family and are involved in the regulation of cancer-associated phenotypes [25].

Ischemic injury has been thought of as a type of common pathological pathway associated with many retinal diseases, such as retinopathy of prematurity, diabetic retinopathy, acute glaucoma, and vein occlusion. Normally, ischemic injury results in neuronal cell degeneration, particularly in retinal ganglion cells, a contributing factor for visual impairment and blindness [26–29]. In previously described model of ischemia-reperfusion (IR) induced injury of the retina, degeneration of retinal ganglion cells occurred in two phases. The first phase occurred within 24 hours (24 h) following injury, and the second phase occurred over the course of several days [30]. We had constructed miRNA-mediated mRNA regulatory networks associated with early and late points following IR-injury of the neuronal retina [31]. In this study, we were reporting a mediation model to examine the roles played by miRNA and TF on gene regulation to complement our previously reported studies [32]. To the best of our knowledge, this study is the first to model miRNA-TF-mRNA interactions in the context of an IR-injury in the retina with mediation analysis. Our goal is to develop a novel approach to characterize the regulatory events in ischemic injury.

## Materials and methods

An extensive miRNA profiling and mRNA profiling of two public datasets, GSE43671 and GSE61072 from the Gene Expression Omnibus (GEO) data repository were used for this study. The two datasets were drawn from a rat model whose intra-ocular eye pressure was increased to reduce blood flow for 60 minutes and then allowed to re-perfuse, for 24h or 7d respectively. The mRNAs array-data were collected at time points of 0h, 24h and 7 days post IR-injury; miRNA-arrays were collected at five time points: 0h, 2h, 24h, 48h and 7d post IR-injury. The mRNA expression data at 2h and at 48h were imputed using a simple least square method [33–35] and paired with equivalent-timed miRNA array data. Agilent single color microarrays were used to quantify the paired transcriptional profiles of miRNA and mRNA expression. Raw data of mRNA and miRNA were imported to GeneSpring (GX 11.1) and normalized. Normalization was performed using a per-chip 75 percentile method that normalizes each chip on its 75 percentile, allowing comparison among chips. Then a per-gene on median normalization was performed, which normalized the expression of every gene on its median among the samples. The miRNA-mRNA expression data whose expressions were altered two or more times (absolute fold-change≥2, and corrected P-value calculated by Benjamini-Hochberg procedure of ≤0.05) in injured versus sham control animals were used as differentially expressed miRNA-mRNA at 0h, 24h, and 7d only as these time points mark the start, apex, and end of the ischemic condition between miRNA and mRNA. Genes with multiple probe ids, had their expression value of the first listed probe id used. Differentially expressed mRNAs and microRNAs were determined as an altered expression at an absolute fold change ≥ 2 and corrected p-value ≤ 0.05 compared to control samples.

**Fig 1:**
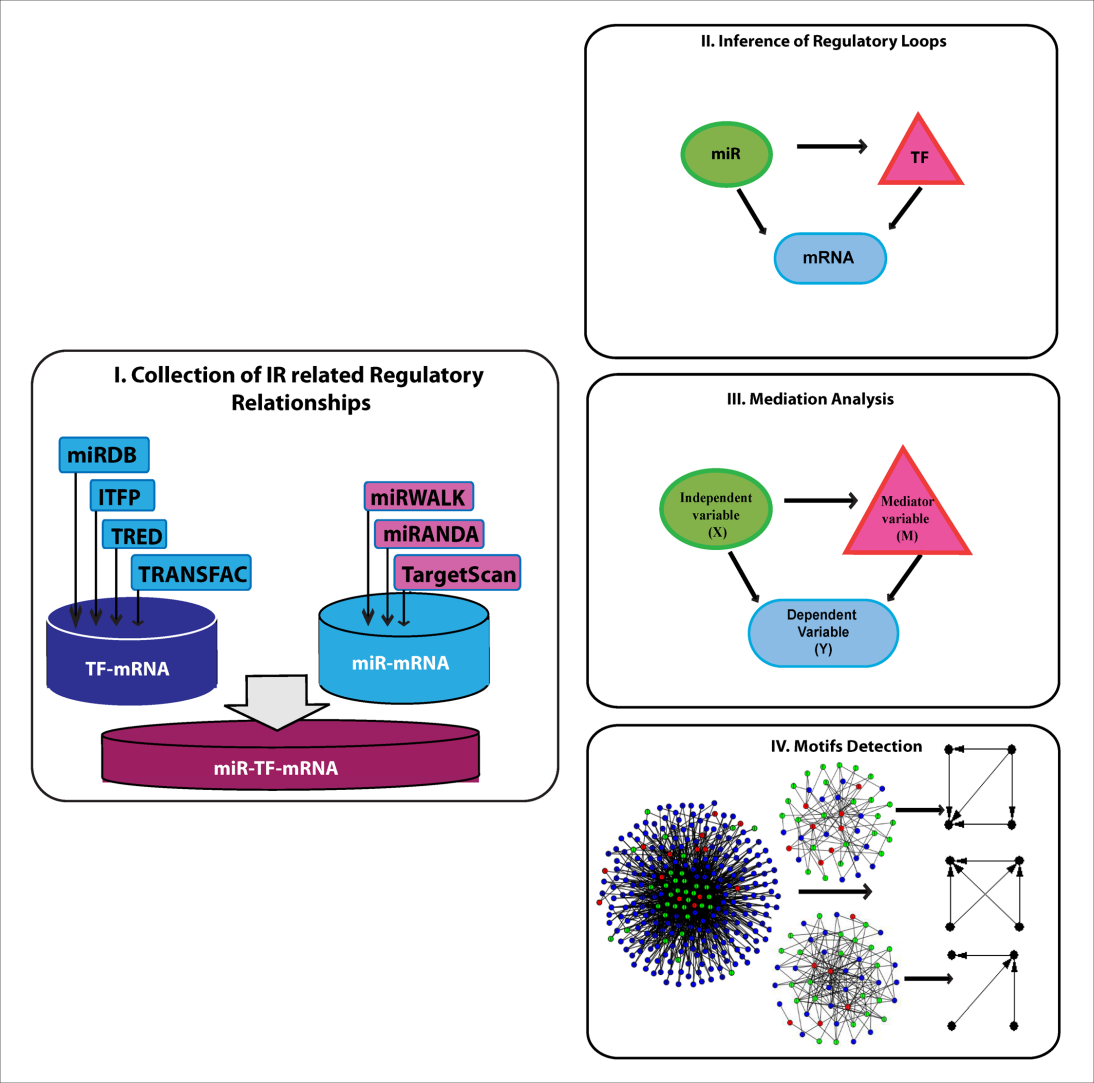
The workflow for inference of the IR- injury mediated regulatory loops. Step I: IR-related miRNAs, TFs, and mRNAs were collected from the experimental mRNA- and miRNA-arrays produced in our laboratory. These represent the altered expression values of the three elements detected at five different time points during ischemia-reperfusion injury. TF-mRNA pairs, miRNA-mRNA pairs, and miRNA-TF pairs were constructed with the aid of external databases and/or software. Step II: The paired constructs were used to build three closed loop-motifs interconnected by three edges. Step III: The closed loops were subjected to mediation analysis resulting in three classes of mediated loops. Step IV: Mediated loops were subjected to motif detection analysis to identify significant regulatory motifs.

The predicted targets of miRNA were obtained from four major public databases, including MiRanda prediction database (August 2010 release) for conserved miRNAs with good mirSVR and the non-conserved miRNAs with good mirSVRs [36,37]. The mirSVR score is a real number computed by machine learning method for ranking microRNA target sites by a down-regulation score. It utilizes prediction rules such as seed-site pairing, site context, free-energy, and conservation. The lower (negative) is the mirSVR score, the better is the prediction. Other microRNAs target sources were TargetScan prediction database (release 6.2) [38], miRWALK prediction database (March 2011 release) [39], and miRTarBase (release 4.5) and its validated target gene database [40]. The latter is an experimentally validated microRNA-target interactions database. Predicted target genes of known transcription factors in rats were collected from several online databases, including ITFP [41], PAZAR [42–43], and TRED [44–45]. Additionally, experimentally validated and predicted TF-mRNA pairs were collected from the commercial database TRANSFAC (professional release 2014) [46] using the Match analysis tool [47], The Match analysis tool was set to investigate the promoter regions of mRNA data (5 kb upstream). To minimize false positives as well as false negatives, only pairs of transcription factors and genes with the highest matrix score (0.8) were collected. Genes unknown to TRANSFAC were re-analyzed with the aid of Match, using either different aliases (gene symbol or RefSeq ID), or using the promoter sequence of the gene as found at the UCSC table browser [48]. We compiled a comprehensive set of all transcription factors with their target genes as reported in those databases and used it to identify TFs in the IR related mRNA data at 0h, 24h, 7d, respectively (Fig 1-I). Querying our mRNA, and microRNA databases, we identified 4218, and 919 regulatory loops at 24h and 7d, respectively.

### Inference of closed regulatory loop motifs

**Fig 2:**
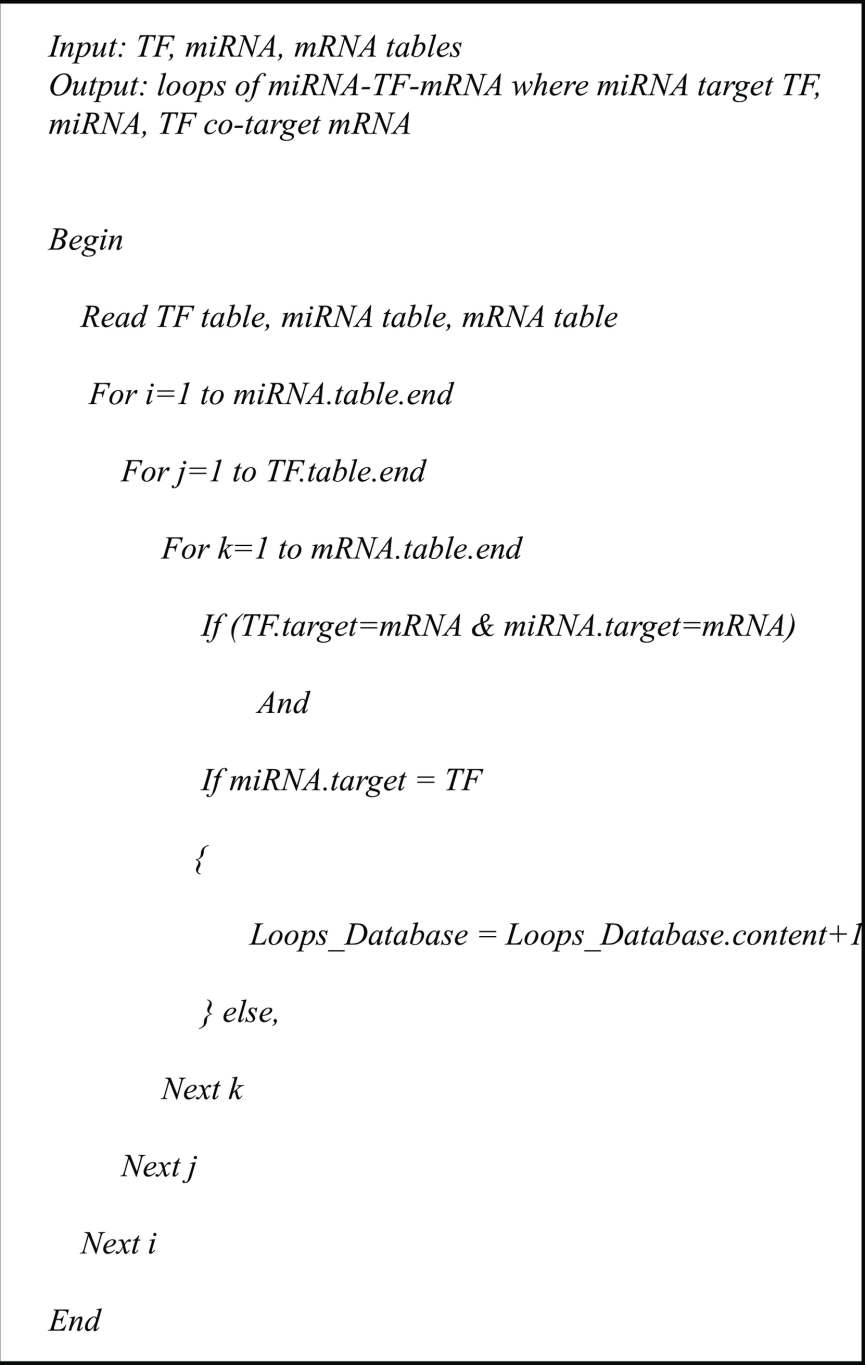
The Pseudo code for inference of closed regulatory loops. Three tables are given as inputs for the algorithm, a transcription factors table TF, a microRNA table miRNAs, and a mRNA table mRNA. If a gene in the mRNA table is a common target by an miRNA and a TF in the miRNA and the TF table respectively, and synchronously the TF is a target for the miRNA, then the triple of miRNA, TF, mRNA is marked as a potential loop and inserted in the Loops_Database repository. Otherwise another gene in the mRNA table should be considered. Previous steps continue until consuming all genes in mRNA table.

Throughout this study, we have used the terms loop and motif interchangeably to indicate regulatory loop motifs. Different patterns of loops can be inferred from these datasets. For simplicity, we restricted our inference to loops where the miRNA targeted a TF and both co-regulated the expression of a co-targeted gene, hence forming a closed triangular loop (Fig 1-II). According to this setting, closed regulatory loops were identified by querying our comprehensive database (Fig 2). The resulting set of loops was mapped to edges and nodes, where each loop was represented by three edges (miRNA-TF, miRNA-mRNA, and TF-mRNA) and three nodes,(miRNA, TF, and mRNA) without regard to direction of interaction. A total of 4,218 and 957 regulatory loops was inferred at 24 h and 7d post-IR period time points respectively. Statistical assessment of individual loops was done by examining the linear and nonlinear correlation between each loop’s three edges, using both Pearson correlation (ρ) [49] and distance correlation (DC) [50]. A nonlinear dependency between some molecules was recently reported [51], which motivated us to consider nonlinear correlation. Only loops with all three significantly correlated edges (p-value≤0.05) were considered for further analyses. Correlation of edges is calculated by the distance correlation method using R package (Energy). The significance of correlation was assessed using function dcor.ttest in the same package. This function applies a nonparametric t-test of multivariate independence with a distribution that is approximately Student t with n (n−3)/2−1 degrees of freedom, and for n ≥ 10 the statistic is approximately distributed as standard normal. The correlation test reduced our number of loops to 2,681 and 699 closed loops at 24 h and 7d post-IR, respectively.

### Unraveling the mediation mechanism

**Fig 3:**
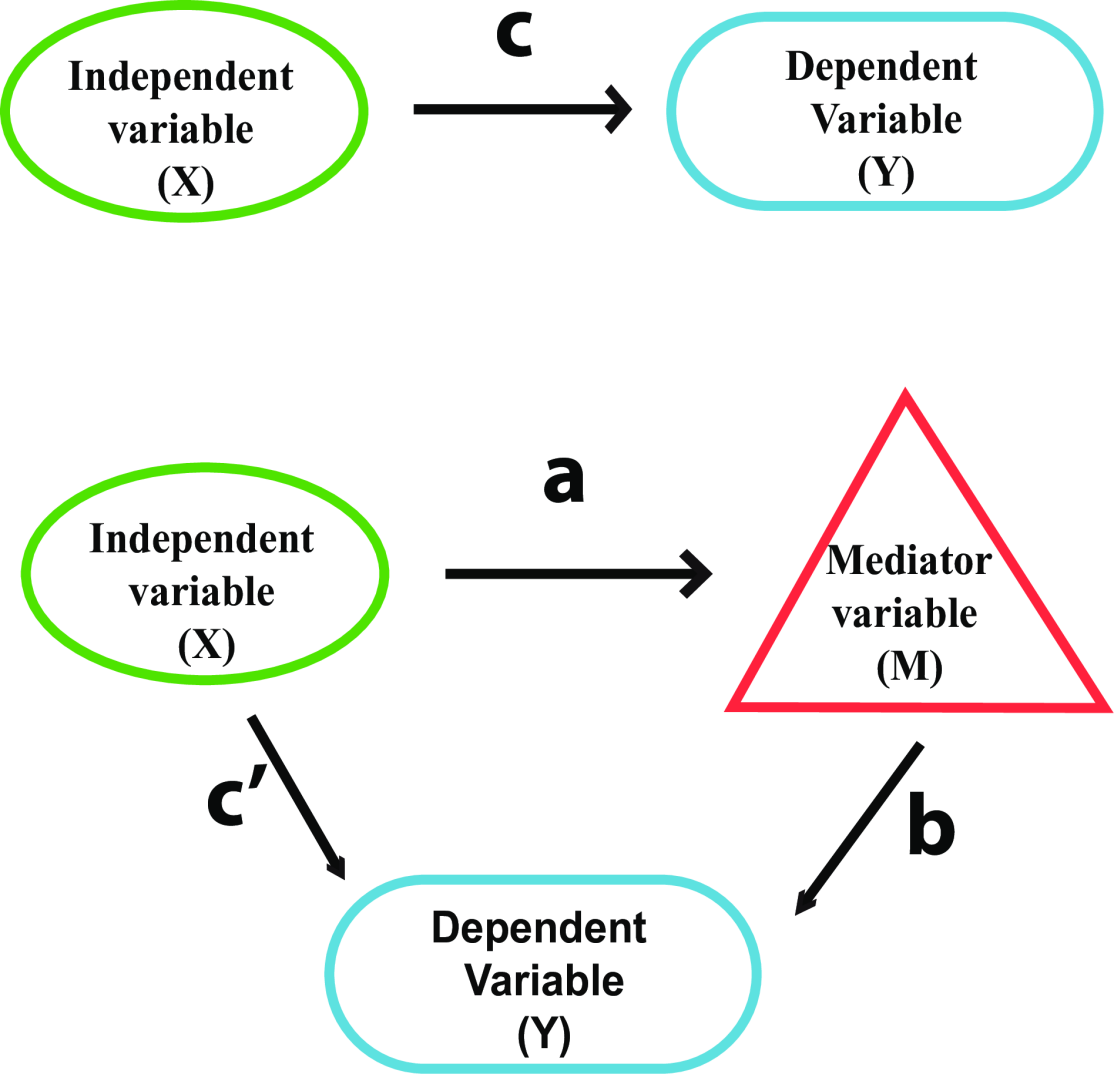
The single variable mediator model. A simple mediation model composed of two variables, X and Y, where Y is dependent on X. The upper diagram illustrates the effect c from X to variable Y in the absence of any additional variables. The lower diagram illustrates how c is split into a, b when a mediator variable is introduced between X, and Y. In this case, effect a is along the X-M path, and effect b is along M-Y path. The split effect will in turn change c into an updated effect cʹ along the X-Y path.

Mediation analysis aims to uncover causal pathways transmitted from causes to effects [52]. It is a model applied [to] systems in which the effect of an independent variable (X) on a dependent variable (Y) is transmitted through a third intervening or mediating variable (M) [53]. This model coincides perfectly with our inferred closed loops (Fig 1-III). The simplest mediation is manifested by a single mediator variable as opposed to multiple mediator variables. The total effect is the entire effect of variable X on Y in the presence of M. When M exists between X and Y, then the effect that is delegated by X to Y through M is called a mediated effect. In a single mediator model (Fig 3), the upper diagram represents the effect of X on Y, and the lower diagram represents the mediated effect of X on Y through M. Mathematically, we can represent these two path diagrams using the regression Eqs in (1), (2), and (3):

$$Y=\beta_{1}+cX+e_{1}$$ (1)

$$M=\beta_{2}+aX+e_{2}$$ (2)

$$Y=\beta_{3}+c\prime X+bM+e_{3}$$ (3)

Where:

Eq (1) is the effect of X on Y in the absence of M, represented by c;
Eq (2) is the effect of X on M, represented by a;
Eq (3) is the effect of X on Y in the presence of M, and it is composed of the effect of X on Y adjusted for M and represented by cʹ, and the effect of M on Y, adjusted for X and represented by b;
β1, β2, and β3 are the intercepts, and e1, e2, and e3 are error terms.

The mediated effect is equal to a*b. The effect of X on Y that does not pass through M is the direct effect of cʹ. Models where cʹ is zero are called completely-mediated models, and models where cʹ is not zero are called partially mediated models. In ordinary-least-squares regression, the total effect is given by Eq 4 while the mediation effect confidence limit and standard error are given by Eqs 5 and 6, respectively.

c=ab+c′ (4)

$$\hat{a}\hat{b}\pm z_{1-w/2}*\;\sigma_{\hat{a}\hat{b}}$$ (5)

$$\sigma_{\hat{a}\hat{b}}=\sqrt{\sigma_{\hat{a}}^{2}{\hat{b}}^{2}+\sigma_{\hat{b}}^{2}{\hat{a}}^{2}}$$ (6)

**Fig 4:**
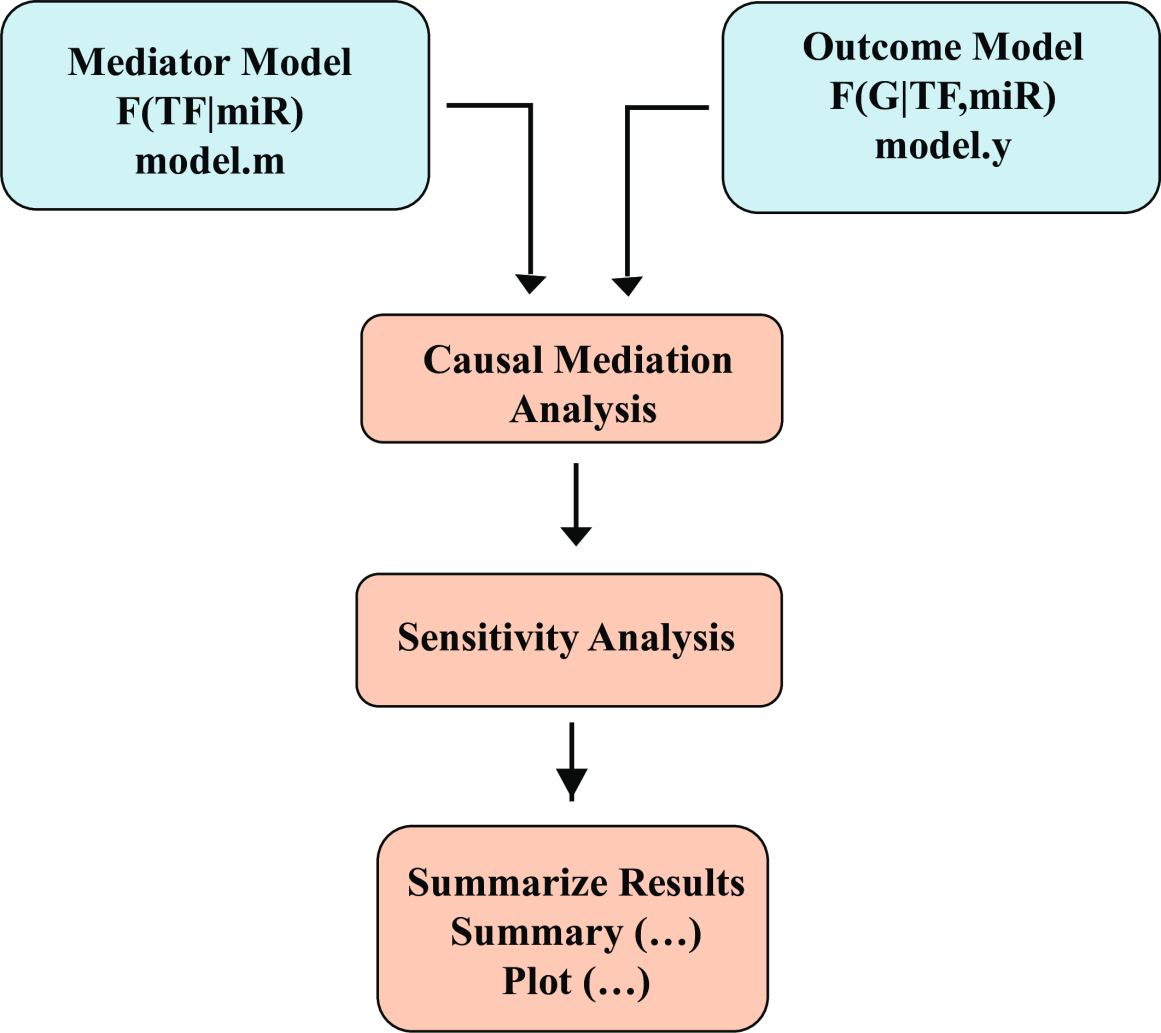
The model-based approach of the causal mediation analysis. At the beginning, two regression models were constructed and fitted separately: model.m and model.y, where model.m modeled the influence dictated by miRNA on TF and model.y modeld the influence dictated by both miRNA and TF on mRNA. The two models were then processed by the causal mediation function. A sensitivity analysis was followed to measure the significance of the model and to plot its summary results.

We fit the linear regression Eqs (1),(2),(3) then followed them by estimating the mediation effects from these models using the standard procedure for analyzing causal mechanisms. A variety of parametric and semi-parametric models can be used to estimate the average causal mediation effect. The core of these modeling approaches is the sequential ignorability assumption for point identification [54], which simply means that the effects of the unobserved factors and missing data can be ignored. According to Imai et al. [55], this assumption provides a general purpose algorithm for estimating the mediation effect. Using the model-based approach (Fig 4) [56–57], we estimated the causal mediation effect in the closed loops in two steps. First, we specified two statistical models, the mediator model M (transcription factor) under treatment T (microRNA) and the outcome model Y (gene) under mediator M and treatment T. Next, the two models were fitted separately and considered as inputs to the mediation algorithm. Since our loops bear linear and nonlinear correlations, we used a linear regression model for linear correlations and a multivariate nonlinear regression model represented by a series of successive cubic regression splines basis defined by three sized sets of knots spread evenly through the covariate values for nonlinear correlations. To calculate the uncertainty estimates associated with the mediation effect, we adjusted the mediation function to use nonparametric bootstrap simulation with a default number of 1000 simulations for the linear correlations and 1000 for the nonlinear correlations. The main outputs from the mediation function were total effect (TE), average causal mediation effect (ACME), and average direct effect (ADE), where TE, ACME, ADE are formulated as:

TE=a*b+c′;

$$ACME=c-c^{\prime }=a*b;$$

$$ADE=c^{\prime }$$

An evaluation of the confidence of the mediation model was achieved by sensitivity analysis for the output. This analysis was necessary to answer questions such as whether the dependent variable expression level -mRNA- deviated from expectations. If so, what would the mediated effect be. The complete results from applying the mediate function for the 24h and 7d post ischemic loops with their associated P-values and confidence intervals are listed in the supporting information S1 File.

## Results

**Fig 5:**
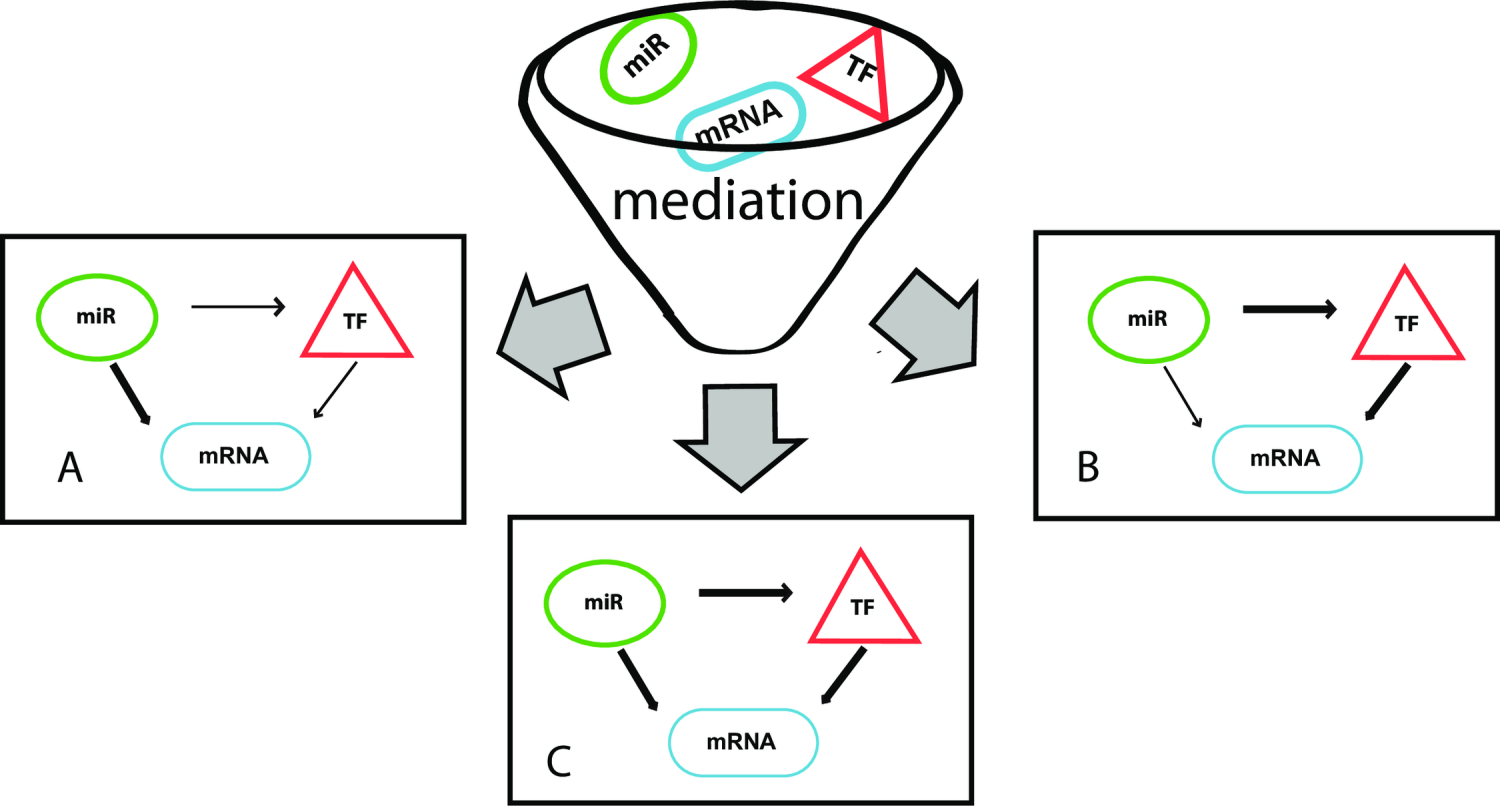
Classification of the closed regulatory loops by mediation analysis. Closed regulatory loops are classified into three main classes: A) The mediated loops by TFs, where all influence on mRNA originates from miRNA delegating its entire influence to the TFs and is represented by M_T_ in the study; B) The mediated loops by miRNAs, where all influence on mRNA originates directly from miRNAs and is represented by M_M_ in the study; C) The mediated loops by TFs and miRNAs together, where all influence on mRNA originates from both miRNAs and TFs and is represented by M_TM_ in the study.

The mediation analysis identified three classes of loops: mediated loops by TFs, mediated loops by miRNAs, and co-mediated loops by both TFs and miRNAs. For simplicity, we will refer to these as class M_T_, M_M_, and M_TM_ loops (Fig 5). The main concern of this study is characterizing these three types of loops, namely, the fraction of loops mediated by TFs alone, the fraction of loops mediated by miRNAs alone, and the fraction of loops mediated by both TFs, and miRNAs at 24h, and 7d IR respectively.

At 24h, the numbers of loops in classes M_T_, M_M_ and M_TM_ were 899, 463, and 472, respectively. In contrast to 24h, the 7d had 216 loops of M_T_, 99 loops of M_M,_ and 220 loops of M_TM_. In total, the mediation analysis explains 1,834 (69%) loops from the 2,681 significant loops at 24h, and 553 (76%) loops from the 699 significant loops at 7d respectively (Table 1). Since transcription factors can work as inhibitors or activators, at 24h class M_T_ loops were further classified into loops with upregulated TFs and genes versus loops with downregulated TFs and genes respectively (Table 2).

**Table 1:** Number of mediated loops per time point per loop class.

Post-IR time point	Loops mediated by TFs only (ACME)	Loops mediated by miRNAs only (ADE)	Loops mediated by both miRNA+TF (ADE+ACME)	Total
24h	899	463	472	1834
7d	216	99	220	553

Number of significant mediated loops (p value ≤ 0.05) with Average Causal Mediation Effect (ACME), Average Direct Effect (ADE), and both (ADE and ACME) in early (24h) and late (7d) post-ischemic time points.

**Table 2:** Classification of mediated loops by TFs per time point.

M_T_: Loops mediated by TF (ACME)			
Post-IR time point	Loops with upregulated TF and TG	Loops with downregulated TF and TG	Total
24h	138	348	486
7d	185	0	185

Number of loops mediated by TFs M_T_(ACME p value ≤ 0.05) in early and late post-ischemic time points. Listed are the numbers of loops, where the expression of the TFs and their target genes (TG) change in the same direction (either both upregulated or both downregulated).

Unlike TFs, miRNAs normally downregulate genes, and hence class M_M_ loops were further classified into loops with upregulated genes and downregulated miRNAs versus loops with downregulated genes and upregulated miRNAs (Table 3). Class M_TM_ involved an influence from miRNAs as well as an influence from TFs, and it is possible for both influences to agree or to differ (Table 4). Therefore, class T_TM_ contained two interesting patterns. The first pattern corresponded to loops with similar ADE and ACME signs, and hence were supporting each other in influencing the gene (Table 4). The second pattern corresponded to loops with different ADE and ACME signs and hence were opposing each other in influencing the gene.

**Table 3:** Classification of mediated loops by miRNAs per time point.

M_M_:Loops mediated by miRNAs (ADE)			
Post-IR time point	Loops with downregulated miRNA and upregulated TG	Loops with upregulated miRNA and downregulated TG	Total
24h	94	146	240
7d	23	11	34

Number of loops mediated by miRNAs M_M_(ADE p value ≤ 0.05) in early and late post-ischemic time points. Listed are the numbers of loops where the expression of the miRNAs and their target genes (TG) changed in opposite directions (miRNA was upregulated and TG was downregulated or miRNA was downregulated and TG was upregulated).

**Table 4:** Classification of mediated loops by both miRNAs and TFs M_TM_ based on signs of ACME and ADME per time point.

M_TM_:Loops mediated by miRNAs (ADE) and TFs(ACME)			
Post-IR time point	Loops with opposing ADE and ACME	Loops with supporting ADE and ACME	Total
24h	216	256	472
7d	142	78	220

Number of loops mediated by miRNAs and TFs M_TM_ (ADE and ACME, p-Value ≤ 0.05) for early and late post-ischemic time points. Listed are the numbers of loops where ADE opposed ACME as well as the numbers of loops where ADE supported ACME.

These two patterns were further classified into loops where the target gene regulation followed either ADE or ACME or ADE and ACME together (Table 5, Table 6).

**Table 5:** Classification of mediated loops by both miRNAs and TFs based on target gene regulation per time point.

Loops with opposing ADE and ACME			
Post-IR time point	Loops where TG follows miRNA(ADE)	Loops where TG follows TF(ACME)	Total
24h	56	160	216
7d	66	76	142

Number of loops mediated by miRNAs and TFs (ADE and ACME, p-Value ≤ 0.05) for early and late post-ischemic time points, where ADE and ACME values changed in different directions (loops with opposing ADE and ACME). The mediated loops are divided to two categories: 1) Loops in which the fold change (FC) of the target gene (TG) followed the direction of the FC of the miRNAs and 2) Loops in which the FC of the TG followed the direction of the FC of the TFs. The numbers of loops in each category are listed.

**Table 6:** Classification of mediated loops by both miRNAs and TFs based on agreement of ACME, ADE, with target gene regulation per time point.

Loops with supporting ADE and ACME			
Post-IR time point	Loops where TG follows miRNA and TF(ADE, ACME)	Loops where TG opposes miRNA and TF(ADE,ACME)	Total
24h	80	176	256
7d	65	13	78

Number of loops mediated by miRNAs and TFs (ADE and ACME, p-Value ≤ 0.05) for early and late post-ischemic time points, where ADE and ACME values changed in the same direction (loops with supporting ADE and ACME). These mediated loops are divided in two categories: 1) Loops in which the fold change (FC) of the target gene (TG) followed the direction of the FC of the miRNA and TF 2) Loops, in which the FC of the TG opposed the direction of the FC of the miRNA and TF. The number of loops in each category is listed.

The highest ACME and ADE values associated with each class of loops at 24h and 7d post ischemic are listed in Table 7. For the M_M_ class at 24h, miR-532-5p and miR-338* were associated with the highest and lowest ADE while at 7d, *miR-495* was the sole miRNA associated with both the highest and lowest ADE. In the M_T_ class, *Gnb2*, *Stat1* were the TFs associated with highest and lowest ACME at 24h, while *Stat1* was the only TF involved with the highest as well as the lowest ACME at 7d. In the M_TM_ class at 24h, the pair *miR-758*, *Stat1* was associated with highest and lowest ADE while the pairs *miR-185*, *Jun*, *and miR-297*, *Maf* were associated with the highest and lowest ACME respectively. On the other hand, at 7d, the pair *miR-483*, *Stat1* and *miR-223*, *Lef1* was associated with highest and lowest ADE respectively while the pair *miR-483*, *Stat1* and *miR-346*, *Bcl6* appeared with the highest and lowest ACME respectively.

**Table 7:** The highest and lowest ACME and ADE per time point per loop class.

Loops with top ACME and ADE values					
Post-IR time point	Loop Type	Highest ACME	Lowest ACME	Highest ADE	Lowest ADE
24h	M_T_	89.91	-139.51	17.23	-12.57
	M_M_	3.81	-8.96	17.32	-20.18
	M_TM_	108.23	-35.5	11.93	-18.86
7d	M_T_	80.57	-18.09	9.6	-6.97
	M_M_	6.78	-2.02	9.69	-7.25
	M_TM_	195.62	-31.84	10.92	-25.5

The highest and lowest ACME and ADE values associated with each class of loops at both post-ischemic time points: M_T_: Loops mediated by TFs, M_M_: Loops mediated by miRNAs, and M_TM_: Loops co-mediated by miRNAs and TFs.

## Discussion

**Fig 6:**
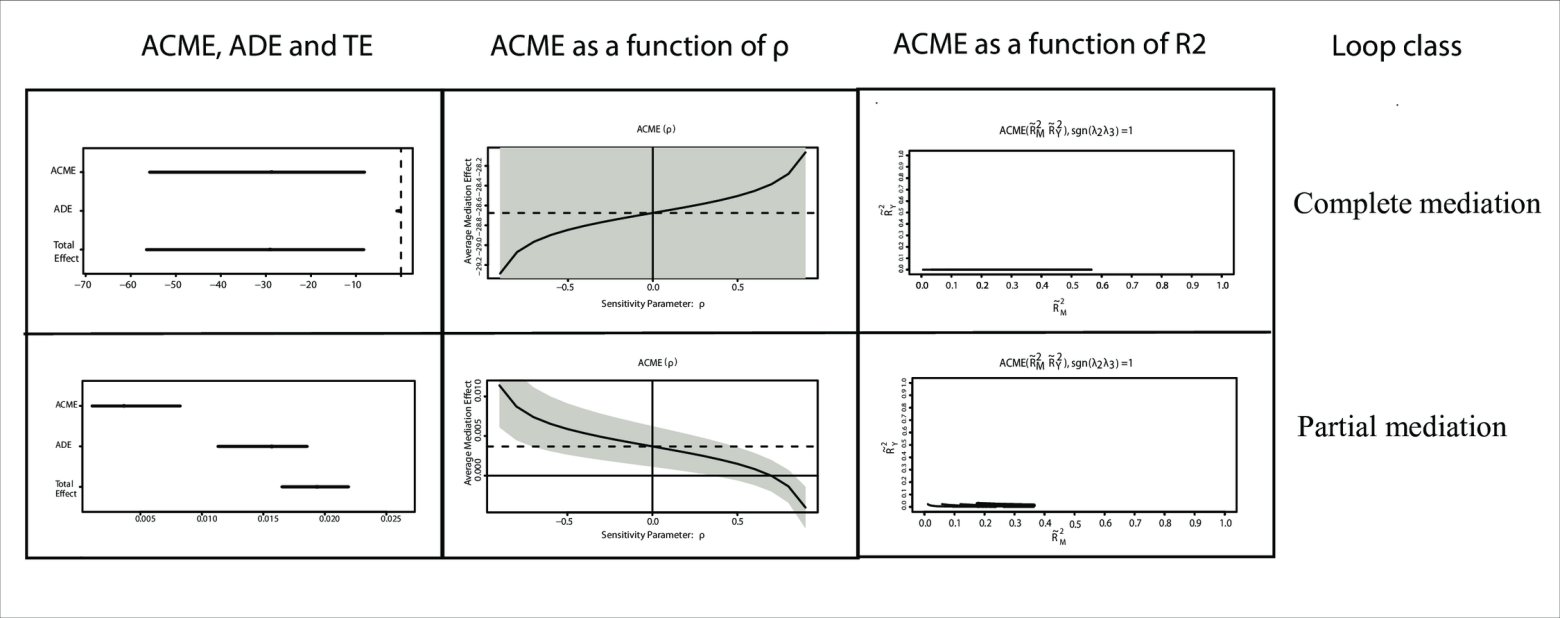
ACME and ADE graphical plots for some exemplary regulatory loops at 24h IR. A dashboard showing graphical plots associated with two types of loops at 24h of IR, partial mediation and complete mediation loop respectively. Each row displays three plots. The left plot shows the average causal mediation effect ACME, average direct effect ADE, and total effect for the particular type of loop on right margin of the plots. The dashed horizontal line represents the estimated mediation effect under the sequential ignorability assumption. The middle and right plots are the sensitivity analysis plots as a function of the standard deviation of the ACME ρ and the mean square error R^2^ respectively. ${\tilde{R}}_{M}^{2}$ is the square of the correlation between independent variables(M→X), while ${\tilde{R}}_{Y}^{2}$ is the square of the correlation between dependent and independent variables(Y→X,M).

Two important outputs of the mediation analysis were the values of the ACME and the ADE. According to [58], the ACME is identified by comparing the ADE before and after introducing the mediator variable to the system. If the ADE disappears or weakens in value after the mediator variable has been introduced, this alteration indicates that the mediator variable has a role and the ACME is to be estimated. If the ADE does not disappear or weaken, then a partial mediation occurs, where the independent and the mediator variables share control of the dependent variable. In a certain model, when no mediation is found, the independent variable could have full control over the dependent variable. The ACME and the ADE for some exemplary loops at 24h are shown (Fig 6).

Both ACME and ADE can have negative or positive values. If ACME and ADE disagree in their signs, this indicates that the mediator variable has an opposite influence to that of the independent variable. If both ACME and ADE agree in their signs, then the mediator variable supports the independent variable. The supporting S2 File lists all the miRNAs and TFs that regulate target genes in both supportive and opposite manners at 24h, and 7d respectively. Some of these regulators have been already associated with different forms of ischemia (e.g. *Creb*[58], *Stat1*[59], *Bcl6*[60], *miR-122*[61], *miR-21*[62], *miR-214*[63], *miR-493*[64]) while others have not (e.g. *Maf*, *Nptx1*, *Lef1*, *miR-290*, *miR-297*, *miR-466*). An interesting observation is that in some regulatory loops, an miRNA-TF combination has an opposing effect on one target gene but a supporting effect on another gene. (*Stat1* and *miR-493* have an opposing effect on *Scfd2*, but a supporting effect on *Dhcr24*.) This phenomenon has been described in the literature for the transcription factor *ATF3* (Activating Transcription Factor 3). Most studies report *ATF3* as a transcriptional repressor. For example, the transcription of tumor suppressor gene *p53* was down-regulated in the *ATF3*-overexpressing cells [65]. Other studies report *ATF3* it as an activator. For example *ATF3* increased the expression of human *IFNGv* [66] as well as of *CD44* and *Bak* [67]. It is thought that *ATF3* combined with different interactive partners can activate genes in-trans [68]. In a manner analogous to system biology, the supporting and opposing regulatory effects on genes are the coherent and incoherent feed backward loops [69]. In coherent loops the regulatory paths have the same overall effect (either activation or repression of the target) similar to the aforementioned supporting effect, while in incoherent loops, the regulatory paths have opposite effects. Therefore, coherent backward loops are suggested in literature to have a bi-stable expression of the miRNAs and TFs involved in the loops. For example, it was reported in the human hematopoietic cells that *mir-233* and *NFI-A* function in a coherent feedback loop to control granulocytic differentiation [69]. In undifferentiated cells, *mir-233* levels are low and *NFI-A* levels are high; however, upon retinoic acid signaling, *mir-233* levels increase and *NFI-A* is repressed, which facilitates differentiation to the myeloid lineage. Therefore, this feedback loop confirms the mutually exclusive expression of *mir-233* and *NFI-A*, thereby generating a bi-stable system (undifferentiated versus differentiated hematopoietic cells). A quite intriguing observation from Table 4 suggests that the opposite sign of ACME and ADE in class M_TM_ loops reveals that the miRNAs and TFs are competing to regulate the target gene in opposite manners [70–71]. The factors that decide the winner are not known, but it is noticeable that more loops with competing miRNAs and TFs occur at 7d than at 24h. The top five positive and negative ADE and ACMEs at each class of loops at 24h and 7d are listed in supporting information S3 File. Generally, ADE had a narrow range intervals at 24h and 7d compared to ACME at the two time points. For example, the ADE values had a range of [-20.18, 17.32] and [-7.25, 9.69] at 24h and 7d respectively. To the contrary of ADE, ACME ranges were [-139.50, 89.91] and [-8.75, 80.57] at 24h, and 7d. This observation reflects the dominant role played by TFs at the two time points. This is consistent with our current understanding of cell death at 24h, which most likely causes an active interaction state between TFs in mediating their target gene. Hence the wide range of ACME at 24h, while at 7d, a dormant interactions trend marked the recession of cell death, and hence the narrow range of ACME [72–73]. Since partial mediated loops imply significant ADE and ACME influence, we therefore list the loops associated with top ADE, as well as top ACME, in S3 File to show both perspectives and ranges of values.

The fact that several loops from S3 File are mediated loops targeting *Hmox1* raises several questions about this gene. However, since we know that *Hmox1* was investigated by many ischemia injury studies and was recently reported to mitigate intestinal ischemic injury reperfusion in rat livers [74–75], we can comprehend why it is a top targeted gene. *Hmox1* may offer new insights about a possible protective function in the context of IR. A more complete understanding of *Hmox1* modifications and the properties that they impart is necessary. Delineating these parameters will provide a clearer picture of the opportunities to modulate *Hmox1* in IR. To validate and to provide a rigorous proof for the mediation results, we used miRWALK database [39] for experimentally validated miRNA targets and TRANSFAC database [46] for experimentally validated TF targets. A complete loop validation requires that the three edges comprising the loop be experimentally tested and validated, which is not found in those databases. However, some partial validations at 24h, 7d respectively are listed in supporting information in the supporting information S4 File.

**Fig 7:**
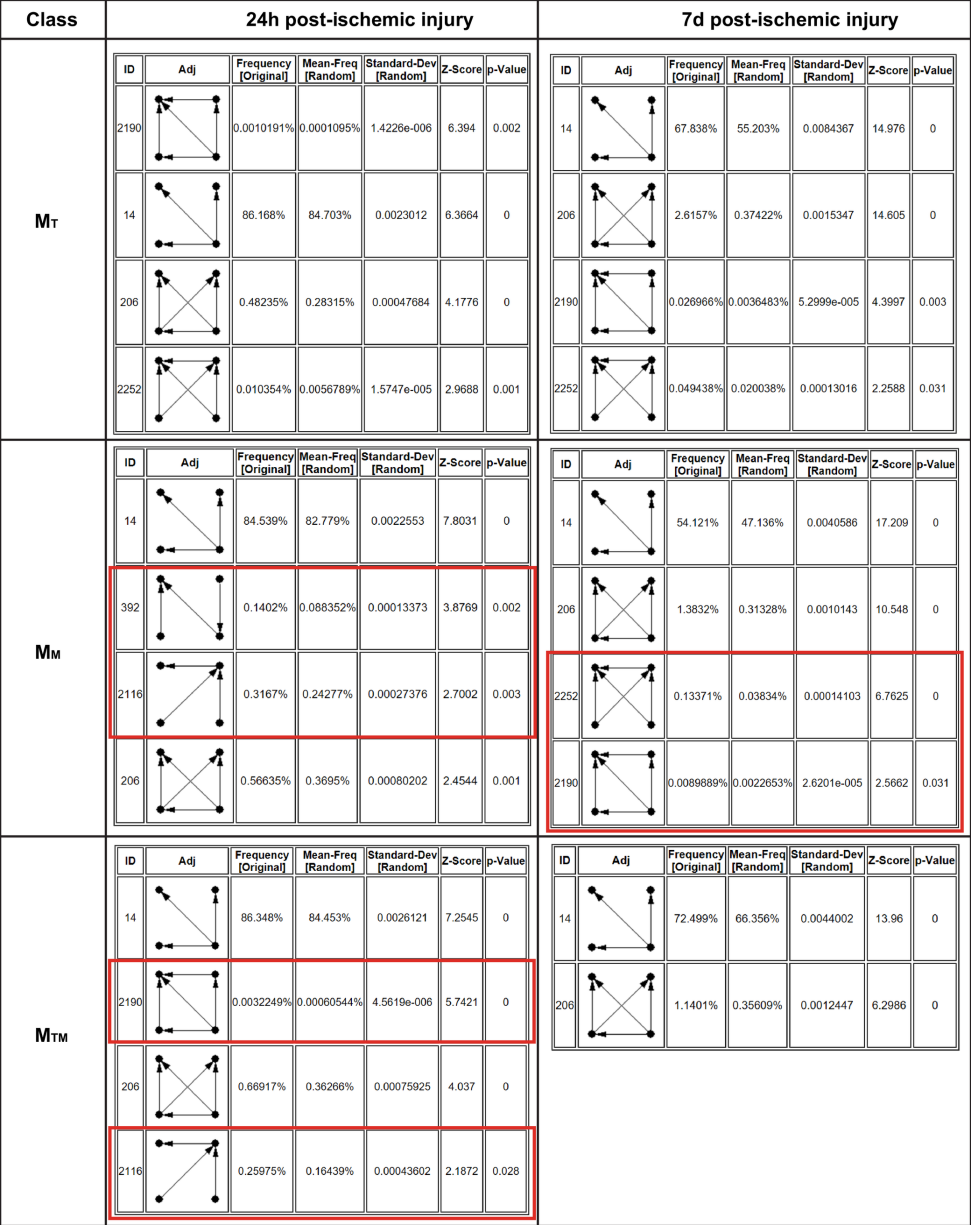
Significant four-node motifs detected by FANMOD exact enumeration algorithm per time point. Four-node significant motifs detected by FANMOD for each class of loops at 24h, and 7d IR. Significance of motifs is determined based on z score ≥ 2 and P-value ≤ 0.05. Unique motifs are surrounded with a red frame in each class.

An important property of networks is so-called network motifs, which are statistically significant recurring subgraphs or patterns. They are significant because they repeat themselves, and their recurring nature indicates that a particular pattern of interactions between vertices may reflect a framework in which particular functions are achieved efficiently. Identifying different network motif types associated with each class of networks is necessary to better understand network biology at each time point. Therefore, the M_T_, M_M_, and M_TM_ class loops at 24h and 7d were searched for statistically significant functional network motifs via the motif detection tools FANMOD [76]. While the computational problem of finding three and four node motifs is tractable, exhaustive enumeration becomes problematic for larger values of nodes, making such analysis impossible [77]. Therefore, FANMOD was adjusted to output significant 4 node motifs, using an exact enumeration algorithm with z-value ≥ 2, p-value ≤ 0.05, and motif frequency occurrence ≥ 5. The output of FANMOD for each class of loops is listed (Fig 7) with a red frame surrounding the unique motifs of each class. Although most of the detected motifs were previously reported in the literature as shown in Table 8, it is important to notice that network motifs do not perform biological functions independently. Instead motifs are interconnected, leading to motif-motif interaction (MMI) pairs [78]. Therefore, it is possible for these motifs to exist in superimposed blocks of the same motif or different motifs.

**Table 8:** Significant network motifs per time point per loop class.

Motif ID	Time Point	Class	Remark	Reference
392	24h	M_M_	_	_
2116	24h	M_M_,M_TM_	_	_
2252	24h|7d	M_T_|M_T_,M_M_	Bi-Feed Forward Loop	[79],[80]
2190	24h|7d	M_T_,M_TM_|M_T_,M_M_	Double Y Motif	[79],[80]
206	24h|7d	M_T_,M_M_,M_TM_	Double Output Motif	[80]
14	24h|7d	M_T_,M_M_,M_TM_	SIM motif	[81],[82]

Significant network motifs in each class of network at 24h and 7d respectively. M_T_: Loops mediated by TFs, M_M_: Loops mediated by miRNAs, and M_TM_: Loops co-mediated by miRNAs and TFs. The motif identification number is displayed under the motif ID. The motif name as found in literature is displayed under Remark. Reference for some of the detected motifs is listed under Reference.

**Fig 8:**
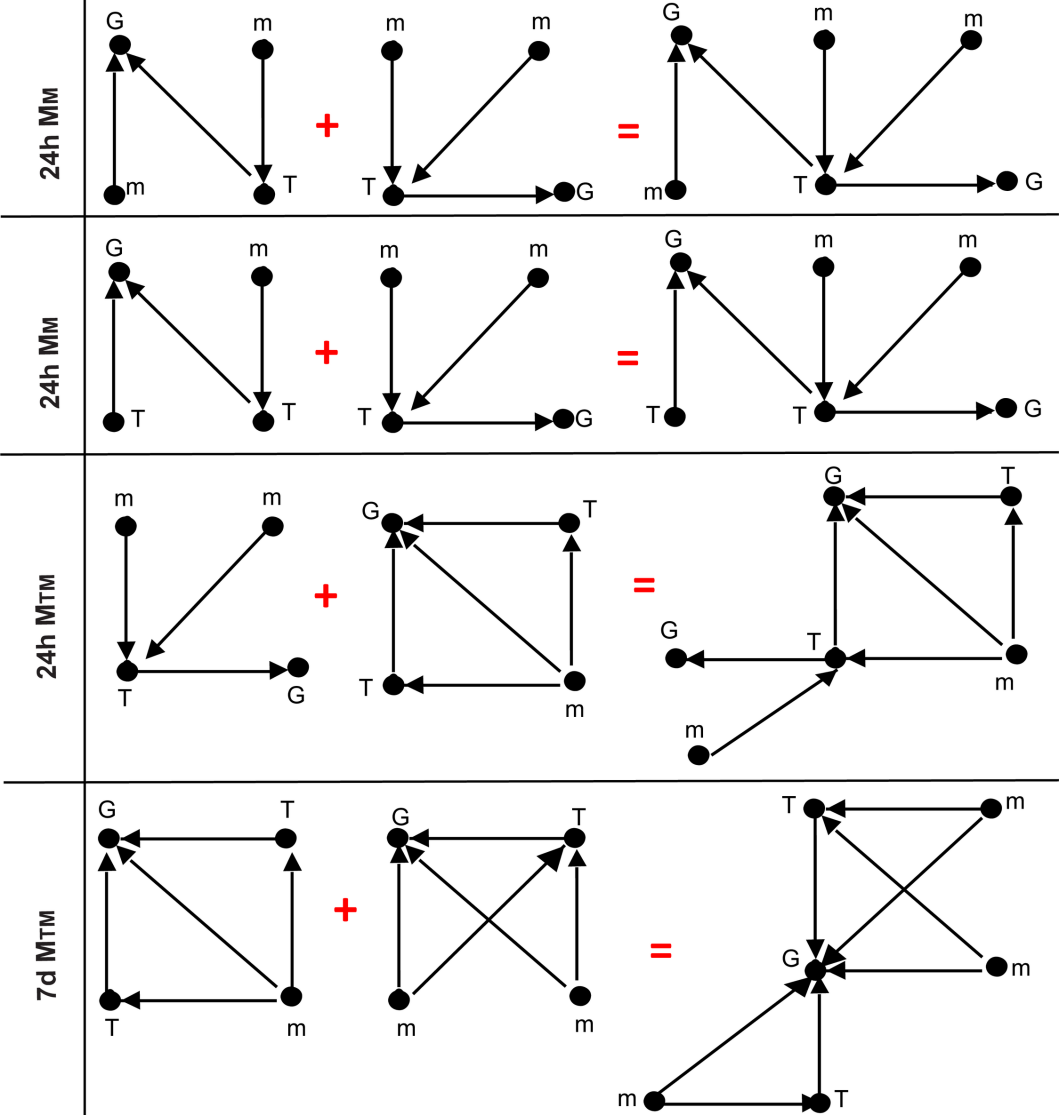
Construction of six-node significant motifs from-four node significant motifs. Two four-node significant motifs at 24h, 7d -IR are assembled to form six-node significant motifs (far right) similar to the six-node significant motifs discovered by FANMOD sampling algorithm. All motifs have z score ≥ 2 and P-value ≤ 0.05.

To get a sense of how these motifs are organized in the networks, we ran FANMOD for six node motifs but with sampling enumeration rather than the exact enumeration. Unlike the exact enumeration, sampling enumeration assigns probabilities for network nodes, and hence it outputs approximate significant network motifs. In spite of the approximation, it does provide insight on possible motif arrangements in the underlying networks. One possible arrangement (Fig 8) from six-node motifs can be composed of the unique four-node motifs in Table 8.

**Fig 9:**
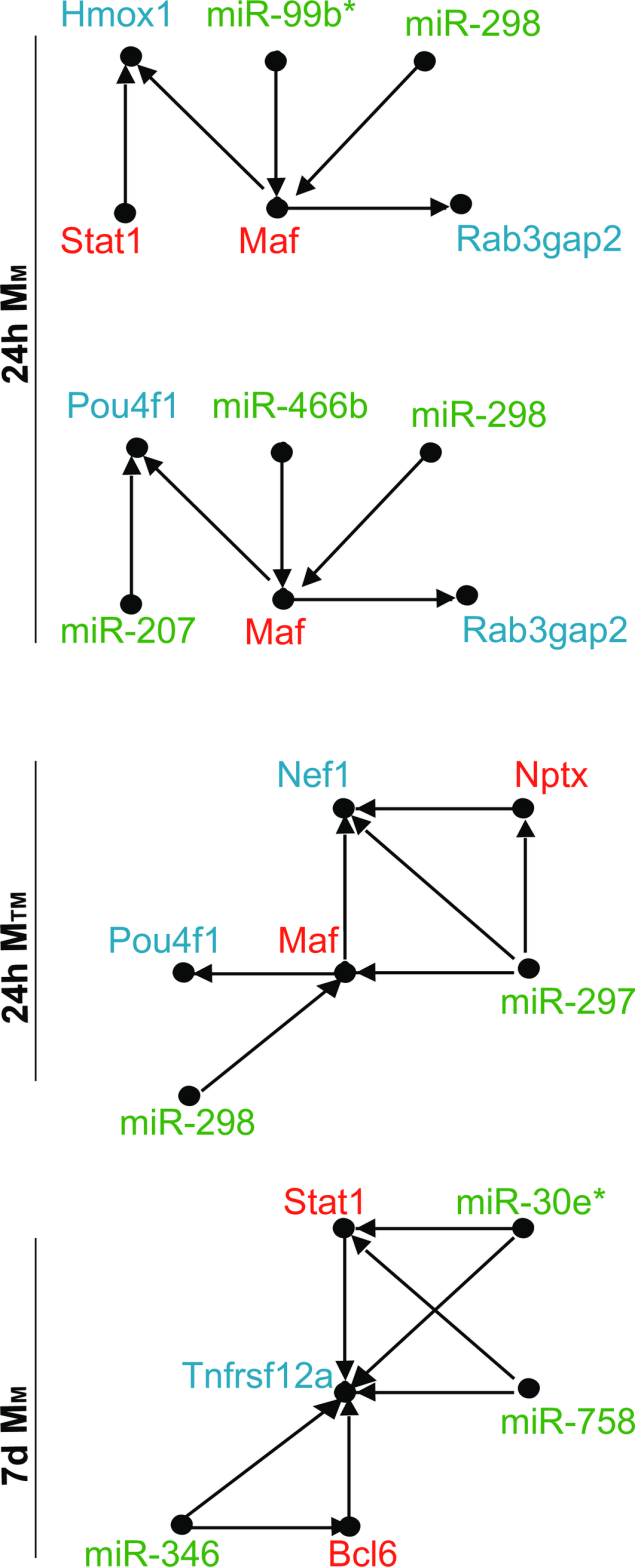
Six-node motifs per time point. Possible six-node motifs arrangements obtained from mapping combined four-node significant motifs at 24h, 7d of IR-injury to the six-node significant motifs in Fig 8. All motifs have z score ≥ 2 and P-value ≤ 0.05.

The motifs were mapped back to the loops in the 24h,7d periods, leading to the loops shown (Fig 9). We argue here that these compound motifs are IR regulatory loops signature that bear important information in deciphering the interplay between miRNAs, TFs, and mRNA in the IR context. We introduce these results for the research community for further consideration with wet lab experiments.

A particular question raised here is whether these results are expected in other conditions, or whether they are ischemic-injury specific results or indeed tissue (retina) specific. Several transcription factors induced in the ischemic brain such as *STAT1*, *MAF* were found to modulate gene expression in the post-ischemic inflammation in humans [83–86]. Additionally, accumulating evidence demonstrates how *FOXO* activation is involved in the mechanisms of ischemic cell death [87–88]. *FOXO* has also been reported in myocardial ischemic injury as well as in ischemic brain [89–90]. Interestingly enough, all three of these TFs were involved in the mediation loops described above.

A complete picture of the ischemic condition requires investigating the cases where the TF is the activator/repressor of both microRNA and target gene. We performed such analysis but did not get a sufficient number of regulatory loops. A database that provides TF-miRNA pairs for the rat’s genome is yet to be developed. We attempted to identify such pairs, however, we were limited to using intragenic miRNAs only. Transcription factors are thought to regulate the transcription of microRNA genes in a manner similar to that of protein-coding genes, that is, by binding to a conventional transcription factor binding site DNA sequences located in or near promoter regions that lie upstream of the microRNA genes [91]. We therefore used the Open Regulatory Annotation database ORegAnno [92] (January 2016 release) for knowledge about Rattus norvegicus transcription factors binding sites. For knowledge about the promoter regions of the microRNA genes, we used both Ensembl [93] (version 69) genome browser and UCSC [94] Rat Jul. 2014 (RGSC 6.0/rn6) genome browser to investigate the promoter regions of all microRNAs genes at 24h and 7d. Both browsers were configured to use Rat genome (Rno 6) and miRBase database (Release 21) for the most updated microRNAs names. The analysis discovered one closed loop of the form of TF-miR-gene at 24h, namely, *Rnf138→rno-miR-207→Creld2*. Applying the mediation analysis on this loop generated a significant direct effect by the TF and an insignificant mediation effect by miRNA. This result confirms the output values from the reverse loop *rno-miR-207→Rnf138→Creld2*, which produced a significant mediation effect by TF and an insignificant direct effect by miRNA. Table 9 lists the ACME and ADE values obtained for both loops.

**Table 9:** ACME and ADE values for the loops: *Rno-miR-207->Rnf138->Creld2* and *Rnf138->Rno-miR-207->Creld2*.

Loop	ADE	Pval_ADE	ACME	Pval_ACME
*miR-207→Rnf138→Creld2*	-0.08	0.48	-1.58	0.0
*Rnf138→miR-207→Creld2*	-0.4	0.0	-0.01	0.45

The upper loop has a significant mediation effect by *Rnf138* (P-value < 0.05) and insignificant direct effect by *miR-207* (P-value > 0.05). Lower loop has an insignificant mediation effect by *miR-207*(P-value > 0.05) and a significant direct effect by *Rnf138*(P-value < 0.05).

In this study, we modeled the tertiary relationship between miRNA-TF-mRNA with a mediation model under the ignorabilty assumption, which overlooks the effect of confounders, i.e. other factors influencing gene regulation. A possible confounder could be an undiscovered higher regulator that contributes to regulating mRNA and interacts with miRNA and TF respectively. Finding such higher regulators may be possible by considering a deep sequencing experiment to capture other small RNAs regulators. Such direction may shed some light on the unverified portion of loops by the mediation model (23% and 21% on 24h, 7d respectively). Lastly, although the type of regulation of target gene is unpredictable by this analysis, the cases where transcription factor[s] intervened between microRNA and gene were revealed.

## Conclusions

In this study, a causal mediation analysis was carried out against ischemic-injury associated regulatory loops derived from rat retinal tissue. The analysis identified three classes of loops at each time point: mediated by TFs only calss, mediated by miRNAs only class, and co-mediated by both TFs and miRNAs class. The latter class is further classified into a subclass where regulators TFs and miRNAs are supporting each other in regulating their co-targeted gene, and another subclass where regulators TFs and miRNAs are opposing each other in regulating their co-targeted gene. Some regulators that have been associated with ischemia and the mediation analysis revealed how they support each other in some cases but oppose each other in other cases include *miR-122*, *Creb1* and *miR-493*, *Stat1*. Other regulators like *miR-297*, *Maf*, and *miR-297*, *Nptx1* have not been associated with ischemia yet. In general, the closed loops were mostly mediated by transcription factors but mediated loops at 7d were very modest next to mediated loops at 24h of IR. Network motif analysis on exemplary loops of each class suggests that these motifs are time point specific IR signatures. A wet laboratory study is needed to confirm these findings.

## Supporting information

S1 File Mediation result and classification of closed regulatory loops at 24h and 7d. A total of eight sheets included. Sheet names are suffixed with “24h” or “7d” to indicate the IR time point and prefixed with “MT”, “MM”, or “MTM” to indicate Mediation by TFs, mediation by miRNAs, and mediation by both TFs, and miRNAs respectively. Sheets “24h”, and “7d” are the full mediation analysis results for all closed regulatory loops at “24h” and “7d” respectively.
(XLSX)

S2 File Supporting and opposing loops at 24h and 7d. A total of four sheets included. Sheet names are suffixed with “24h” or “7d” to indicate the IR time point and prefixed with “supporting”, “opposing” to indicate pairs of miRNAs-TFs that are working together or against each other respectively.
(XLSX)

S3 File Top mediated loops for each class of loops at 24h and 7d. A total of two **s**heets included for 24h, and 7d respectively. Each sheet contains four additional tables listing the top five mediated loops in each class of mediated loops.
(XLSX)

S4 File Validated mediated loops 24h and 7d. Partial validation from miRWALK db. A total of six sheets included. Sheet names are suffixed with “24h” or “7d” to indicate the IR time point and prefixed with “MT”, “MM”, or “MTM” to indicate Mediation by TFs, mediation by miRNAs, and mediation by both TFs, and miRNAs respectively.
(XLSX)
